# The Roles of an Aluminum Underlayer in the Biocompatibility and Mechanical Integrity of Vertically Aligned Carbon Nanotubes for Interfacing with Retinal Neurons

**DOI:** 10.3390/mi11060546

**Published:** 2020-05-28

**Authors:** William J. Watterson, Saba Moslehi, Conor Rowland, Kara M. Zappitelli, Julian H. Smith, David Miller, Julie E. Chouinard, Stephen L. Golledge, Richard P. Taylor, Maria-Thereza Perez, Benjamín J. Alemán

**Affiliations:** 1 Physics Department, University of Oregon, Eugene, OR 97403, USA; william.j.watterson@gmail.com (W.J.W.); sabam@uoregon.edu (S.M.); crowlan2@uoregon.edu (C.R.); karaz@uoregon.edu (K.M.Z.); jhs@uoregon.edu (J.H.S.); dmiller3@uoregon.edu (D.M.); 2 Material Science Institute, University of Oregon, Eugene, OR 97403, USA; 3 Oregon Center for Optical, Molecular and Quantum Science, University of Oregon, Eugene, OR 97403, USA; 4 Center for Advanced Materials Characterization in Oregon, University of Oregon, Eugene, OR 97403, USA; jbarkman@uoregon.edu (J.E.C.); golledge@uoregon.edu (S.L.G.); 5 Department of Clinical Sciences Lund, Division of Ophthalmology, Lund University, SE-221 84 Lund, Sweden; 6 NanoLund, Lund University, SE-221 00 Lund, Sweden

**Keywords:** VACNTs, retinal implants, cell culture, biomaterials, retinal neurons, glia

## Abstract

Retinal implant devices are becoming an increasingly realizable way to improve the vision of patients blinded by photoreceptor degeneration. As an electrode material that can improve restored visual acuity, carbon nanotubes (CNTs) excel due to their nanoscale topography, flexibility, surface chemistry, and double-layer capacitance. If vertically aligned carbon nanotubes (VACNTs) are biocompatible with retinal neurons and mechanically robust, they can further improve visual acuity—most notably in subretinal implants—because they can be patterned into high-aspect-ratio, micrometer-size electrodes. We investigated the role of an aluminum (Al) underlayer beneath an iron (Fe) catalyst layer used in the growth of VACNTs by chemical vapor deposition (CVD). In particular, we cultured dissociated retinal cells for three days in vitro (DIV) on unfunctionalized and oxygen plasma functionalized VACNTs grown from a Fe catalyst (Fe and Fe+Pl preparations, where Pl signifies the plasma functionalization) and an Fe catalyst with an Al underlayer (Al/Fe and Al/Fe+Pl preparations). The addition of the Al layer increased the mechanical integrity of the VACNT interface and enhanced retinal neurite outgrowth over the Fe preparation. Unexpectedly, the extent of neurite outgrowth was significantly greater in the Al/Fe than in the Al/Fe+Pl preparation, suggesting plasma functionalization can negatively impact biocompatibility for some VACNT preparations. Additionally, we show our VACNT growth process for the Al/Fe preparation can support neurite outgrowth for up to 7 DIV. By demonstrating the retinal neuron biocompatibility, mechanical integrity, and pattern control of our VACNTs, this work offers VACNT electrodes as a solution for improving the restored visual acuity provided by modern retinal implants.

## 1. Introduction

Photoreceptor degeneration (i.e., loss of rods and cones) in the retina can lead to blindness in patients with retinitis pigmentosa (RP) or age-related macular degeneration (AMD) [1,2]. Retinal implants are currently restoring vision to these patients by electronically stimulating the remaining healthy portions of the retina. However, the restored visual acuity provided by these implants remains significantly below the acuity required to read text or recognize faces [3,4,5]. One factor that significantly reduces visual acuity is poor signal fidelity and transmission during neuronal stimulation, which is largely dictated by the electrode–neuron interface [6]. Signaling across the interface could be improved through an appropriate choice of material properties [7,8] and electrode geometry [9,10].

An electrode material for neural implants must combine biocompatibility with a demanding set of electrical, chemical, and mechanical properties. Electrically, the electrode must inject sufficiently large currents into the neural tissue, ideally through a purely capacitive means [11]. Chemically, electrode materials must resist degradation in the physiological environment of neural tissue and should support surface functionalization to increase their hydrophilicity, thereby preventing neuronal cell death and stimulating neurite outgrowth [12], and also reduce the voltage threshold needed for neuronal stimulation [13]. Mechanically, they need to be strong and yet flexible to reduce inflammation and glial scarring in the surrounding tissue [14,15,16]. Furthermore, soft materials (i.e., mechanically compliant) [17,18] and materials with rough, textured surfaces [19,20] can enhance neurite outgrowth, elongation rate, and branching.

The electrode geometry is vital to the electrode–neuron interface. Electrodes with vertically oriented high-aspect-ratio features such as grooves [21,22] or nanowires [23] can greatly promote neuron adhesion and outgrowth while reducing the gliotic response [24,25]. By increasing the overall electrode surface area, these features also enhance charge injection. In subretinal implants, high-aspect-ratio electrodes penetrate the inner nuclear layer, thereby reducing the voltage thresholds required to stimulate neighboring bipolar neurons [26,27]. The planar geometry can also enhance neural signaling. For example, relative to rectangles or grids, electrodes featuring fractal geometries are predicted to better stimulate neurons and reduce the pixel size of electrode arrays [9,10,28]. However, to exploit these geometric enhancements, the electrode material must be patternable.

Carbon nanotubes (CNTs) are a patternable electrode material which simultaneously meet all material requirements for neural prosthetics. CNTs are electrically conductive and have a double layer capacitance (~10 mF/cm^2^) [29] that compares favorably to nearly every other material. CNTs can be chemically functionalized to improve neurite outgrowth and branching [30,31]. They are mechanically flexible [32,33,34] yet incredibly strong [35], and their molecular-scale diameter (~1–10 nm) promotes strong adhesion and electrical coupling with neurons [36,37]. Vertically aligned carbon nanotubes (VACNTs) can be patterned and synthesized by CVD to form high-aspect-ratio structures with heights exceeding 500 µm [38], and because neurons adhere strongly to CNTs, patterned CNTs can be used as scaffolds to guide neurite growth [39]. Importantly, the biocompatibility of VACNTs has been examined in vitro with several types of neural tissue, including rat hippocampal and cortical neurons, NOBEC cell line (glial cells), and retinal precursor cells [38,39,40,41,42]. Due to this combination of useful properties, CNTs have been employed in multi-electrode arrays (MEAs) for epiretinal implants [43,44] and in vitro MEA studies where CNTs improved the signal-to-noise ratio, lowered the stimulation threshold, and minimized glial scarring [45].

Despite many promising advances, CNTs have not been widely adopted in neural electrodes because of the structural fragility of CNT films. This fragility is largely caused by poor CNT/substrate adhesion. In the CVD growth of VACNTs where the catalyst (i.e., Fe or nickel (Ni)) is deposited directly onto the substrate, the nanotube films often delaminate and crack [44,45], preventing them from acting as conducting electrodes. Moreover, delamination of CNTs increases the concentration of CNTs dispersed in the culture medium, which can reduce axonal regeneration [46]. A simple approach to strengthen the VACNT/substrate bond is to deposit a thin film of Al between the substrate and the catalyst [47]. While the Al underlayer does greatly reduce cracking and delamination, the neurotoxicity of Al raises the vital and previously unresolved question about the biocompatibility of the Al-stabilized VACNTs. Here, to test this biocompatibility, we seeded dissociated retinal neurons onto VACNTs for several days in vitro (DIV) and measured the extent of neurite outgrowth and the percentage of neurons with outgrowth to infer biocompatibility, as it is well established that neurite outgrowth and cell viability are correlated [48]. We were particularly interested in neurite outgrowth as a measure of cell viability and as a basis for future neurite stimulation purposes. We compared various preparations of CVD VACNTs grown with an Fe catalyst and an Al underlayer, and functionalized with oxygen plasma. We found that the Al layer increased the mechanical integrity of the nanotube film, enhanced retinal neurite outgrowth over the Fe-only preparation, and also supported neurite outgrowth for up to 7 DIV. Unexpectedly, we found that the plasma preparation significantly reduced neurite outgrowth, even though plasma functionalization typically boosts biocompatibility [31]. Moreover, we used our Al-based growth to generate finely patterned, high-aspect-ratio VACNT electrodes with lateral dimensions as small as ~2.5 μm. Our results demonstrate that VACNTs prepared with an Al underlayer are viable electrode candidates for retinal implants.

## 2. Materials and Methods

### 2.1. Carbon Nanotube Growth

Three distinct groups of VACNT forests (consisting of CNTs oriented along their longitudinal axes perpendicular to the substrate) on silicon/silicon dioxide substrates were fabricated. The first group was made of uniform VACNT forests and consisted of four preparations in total, using either a Fe catalyst alone (Fe preparation) or a Fe catalyst on an Al underlayer (Al/Fe preparation). Two additional preparations were created by oxygen plasma functionalization (Fe+Pl and Al/Fe+Pl preparations). Substrates (University Wafer) were ~1 cm^2^ in size with a thermal oxide thickness of 300 nm. Briefly, metals were evaporated onto the entire substrate using a thermal evaporator to a thickness of 4–8 nm for Al and 6–12 nm for Fe. CNT growth was performed by atmospheric pressure CVD in a 2” quartz tube for 5 min at 650 °C in a 2:1 mixture of ethylene (C_2_H_4_) and hydrogen (H_2_) at 200 and 100 SCCM, respectively, in the presence of 600 SCCM flow of argon (Ar). This growth resulted in a VACNT forest covering the entire chip. The uniform VACNTs had a height range of 30–38 µm. To functionalize the surfaces, we exposed the VACNT forests to oxygen plasma in a South Bay Technology PC-2000 Plasma Cleaner at 300 mTorr O_2_ (70 mTorr base pressure) for 5 min at 50 W forward power [49,50,51].

**Figure 1:**
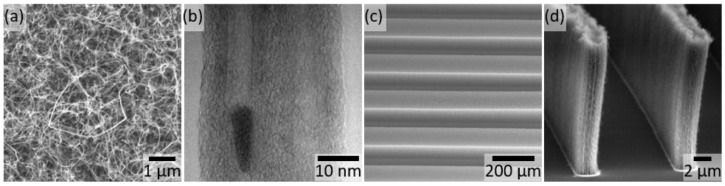
Images of (group one) vertically aligned carbon nanotube (VACNT) forests grown from the Al/Fe catalyst. (**a**) SEM showing the top-down view of uniform VACNT forests. (**b**) TEM showing two aligned CNTs from the Al/Fe preparation. The dark contrast vertical lines are the walls of the multi-walled CNT (MWCNT). The dark object encapsulated inside the leftmost nanotube is likely a catalyst nanoparticle. (**c**) SEM image of (group two) VACNT rows 100 µm in width with 100 µm spacing, imaged at a 30 ° tilt. (**d**) SEM image of (group three) VACNT rows with a 2.5 µm width, 10 µm spacing, and 20 µm height imaged at an 85° tilt.

The second group consisted of patterned rows of VACNTs with a width of 100 µm separated by 100 µm rows of silicon dioxide (Figure 1c). Photolithography techniques were used to create these patterns on a 6 mm × 6 mm area of the substrates. After photoresist lift-off, VACNTs were synthesized using the Al/Fe preparation mentioned above for the first group. The CNT growth time for the second group was 3 min and generated VACNTs with a height range of 21–31 µm. This group was not plasma functionalized. Additionally, to demonstrate the ability to construct high-aspect-ratio electrodes for future high visual acuity subretinal implants, a third group consisting of rows of VACNTs grown using the Al/Fe preparation was synthesized using photolithography to generate patterns with row width and row separation down to 2.5 µm and a height of 20 µm (Figure 1d and Figure S1). The metal layer thicknesses and CVD growth conditions for the second and third groups were similar to those for the Al/Fe preparation uniform forests.

### 2.2. Carbon Nanotube Characterization

VACNT surfaces were characterized using scanning electron microscopy (SEM), transmission electron microscopy (TEM), Raman spectroscopy, X-ray photoelectron spectroscopy (XPS), and contact angle goniometry. Visual characterization of the VACNT structure, topography, and height was carried out using a Zeiss Ultra-55 SEM. TEM imaging of the nanotubes was performed with a FEI Tecnai operated at 80 keV. Raman spectroscopy provided information on the bonding, crystallinity, and defects of the CNTs, and was performed using a WITEC alpha300 Raman microscope with a 532 nm excitation laser. The atomic composition and additional bonding information of VACNTs were obtained by XPS using a Thermo Scientific Escalab 250. XPS is sensitive to the outermost 8–10 nm of the CNT surface. Hydrophobicity of the CNTs was determined by measuring the wetting contact angles (WCAs) with a Ramé–Hart Model 290 goniometer. Additional details for the TEM, Raman spectroscopy, XPS, and contact angle goniometry are provided in the Supplementary Materials (SM).

### 2.3. Dissociated Retinal Cell Cultures

All in vitro studies were performed under the approval of the University of Oregon Institutional Animal Care and Use Committees under protocol 16-04, in compliance with National Institutes of Health guidelines for the care and use of experimental animals.

The four different preparations of the uniform VACNTs (group one) were used to test the effects of these preparations on retinal cells in 3 DIV dissociated retinal cell cultures. The choice of uniform VACNTs eliminated potential complications introduced by patterning the samples. Only preparations where the VACNT mat appeared intact were subsequently used for cell culture experiments.

Retinal cells were obtained from wildtype C57BL/6 mice at postnatal day 4, as previously described [23,24]. Animals were first euthanized, and then whole retinas were dissected from the eyes and placed into Dulbecco’s modified eagle medium (DMEM) with high-glucose, sodium pyruvate, and L-glutamine (Thermo Fisher Scientific). After dissection, 4 retinas were transferred into an enzyme solution and digested for 22.5 min at 37 °C to loosen cell-cell adhesion. The enzyme solution was prepared by combining 3 mL DMEM, 3 mg papain (Worthington Biochemical Corporation), and 0.9 µg L-cysteine (Sigma-Aldrich) and filtering through a 0.22 µm filter (Sarstedt, Newton, NC, USA). After enzyme digestion, the enzyme solution was removed and the retinas were rinsed thoroughly in DMEM. Digested retinas were then placed into 2 mL of the final culture medium. The final culture medium was prepared by mixing 21.34 mL of DMEM, 440 µL of B27 supplement (Sigma-Aldrich, St. Louis, MO, USA), and 220 µL L-glutamine-penicillin-streptomycin (Sigma-Aldrich, St. Louis, MO, USA). The final culture medium plus digested retinas were then mechanically agitated through a rounded Pasteur pipette to break the whole retinas into single cells and cell clusters. Next, 48 mL of DMEM was added to the 2 mL dissociated retina solution and centrifuged at 900 g for 5 min. After centrifugation, the supernatant was removed and the remaining 20 mL of final culture medium was added to the cell pellet. The cells were again mechanically agitated to re-suspend cells throughout the solution. The cell suspension was then passed through a 40 µm cell strainer filter (Fisher Scientific) to remove large cell clumps. Finally, 500 µL of cell suspension containing ~8.5 × 10^5^ cells was carefully plated directly onto a VACNT sample placed in the middle of each well of a 4-well culture plate (Sarstedt, each well 1.9 cm^2^) and cultured for 3 DIV at 37 °C and 5% CO_2_. Cell plating was randomized between the sample preparations (Fe, Al/Fe, Fe + Pl, and Al/Fe + Pl). No chemical functionalization, such as precoating with extracellular matrix (ECM) proteins, was done to the VACNT samples prior to seeding the cells.

The second group of samples (100 µm wide rows of patterned VACNTs grown from an Al/Fe catalyst layer) was used in 3 and 7 DIV retinal cell cultures to examine the longer-term biocompatibility of the best preparation (i.e., Al/Fe preparation—see results section) and to do so using patterned rather than uniform electrodes (which are more relevant for future applications). The culture protocol was the same as explained before. The seeding density on the patterned samples was twice that of the uniform samples, and the cells were incubated for 3 and 7 DIV under the same conditions. Medium changes were performed on the third and fifth days for 7 DIV cultures. The third group of samples (patterned high-aspect-ratio VACNTs grown from an Al/Fe catalyst layer) were not used in cultures for the biocompatibility test and were used only to demonstrate the fabrication and patterning capabilities for future studies.

### 2.4. Immunohistochemistry

Fluorescent labeling of neurons and glia was achieved through dual staining immunohistochemistry. First, the culture was terminated by fixing the cells with 4% paraformaldehyde (PFA) for 30 min. After fixation, the PFA was rinsed off with 1x phosphate buffered solution pH 7.3 (PBS), and then cells were pre-incubated in a PBScomp solution containing 2% donkey normal serum (DNS) and 2% goat normal serum (GNS) (Jackson ImmunoResearch, West Grove, PA, USA) for 1 hour at room temperature. PBScomp was prepared from 1x PBS, 0.25% Triton-X (Sigma-Aldrich) and 1% bovine serum albumin (Sigma-Aldrich). The pre-incubation solution was then removed and the samples were incubated in the primary antibody solution overnight at 4 °C. The primary incubation solution contained PBScomp, 2% DNS, 2% GNS, 1:1500 rabbit anti-β-tubulin III (neuronal marker) (Sigma-Aldrich), and 1:1500 goat anti-glial fibrillary acidic protein (GFAP, a glia marker) (Dako). Next, the primary incubation solution was removed and samples were rinsed again in PBS. They were then incubated in a secondary antibody solution containing PBScomp, 1:400 Alexa Fluor 488 donkey anti-rabbit IgG, and 1:200 Cy3 goat anti-mouse IgG (Jackson ImmunoResearch) for 45 min at room temperature. Afterwards, the secondary antibody solution was removed and samples were rinsed again. Finally, the samples were mounted with Vectashield containing DAPI (DAPI attaches to DNA in the cell nucleus) (Vector Labs).

### 2.5. Epifluorescence Microscopy

Uniform VACNT (group one) samples were imaged using a Nikon Eclipse Ti-U epifluorescence optical microscope with an iXon Ultra 888 EMCCD camera at 20× magnification (Nikon CFI S Plan Fluor objective, NA 0.45). A total of 20 randomly chosen positions on each VACNT sample were imaged to statistically assess cell response on that sample. The field of view (FOV) of each image was 0.20 mm^2^. The second group of samples (100 µm wide rows of patterned VACNTs grown from an Al/Fe catalyst layer) was fully imaged at 20× magnification using a Leica DMi8 inverted fluorescence microscope. Each FOV was 0.44 mm^2^.

### 2.6. Statistical Analysis

Normalized neurite length was examined as the neuron response variable for the different VACNT preparations. To quantify the neurite length, an automated image analysis algorithm based on one previously reported by Wu et al. was developed [52]. The developed algorithm allowed us to measure the total neurite length in each FOV, but not the neurite length per neuron. For each sample, the normalized neurite length (N_CNT_) was calculated by dividing the total neurite length over all FOVs by the available VACNT area. Additionally, the numbers of neurons with and without neurite outgrowth were counted manually for five randomly chosen FOVs per sample (8 samples for each preparation) using the ImageJ Cell Counter plugin. The percentage of neurons bearing a neurite was then calculated for each sample. We tested via Kruskal–Wallis analysis with a post-hoc Dunn’s test against the null hypothesis that the normalized neurite length and percentage of neurons with neurite outgrowth per sample were not dependent on the VACNT preparation for the uniform (group one) samples. For the patterned (group two) samples, the null hypothesis was tested against culture duration instead of CNT preparation. In total, the uniform VACNT samples with 8 per preparation (Fe, Al/Fe, Fe + Pl, and Al/Fe + Pl) were tested across 3 independent 3 DIV cultures, and the patterned samples with 6 at 3 DIV and 11 at 7 DIV were tested across 9 independent cultures.

## 3. Results

The uniform (group one) VACNTs fabricated for the biocompatibility tests were densely packed (Figure 1a,d) and there were no observable differences in CNT nanotopography via SEM imaging between any of the four preparations. Figure 1a shows an example of the top surface of the Al/Fe preparation. Figure S2 columns (a) and (b) show ~3 k× and ~7.5 k× magnification SEM images of the top surface of all four VACNT preparations. Figure S2 column (c) shows a 30° tilted view with ~4 k× magnification of the four preparations. 

**Figure 2:**
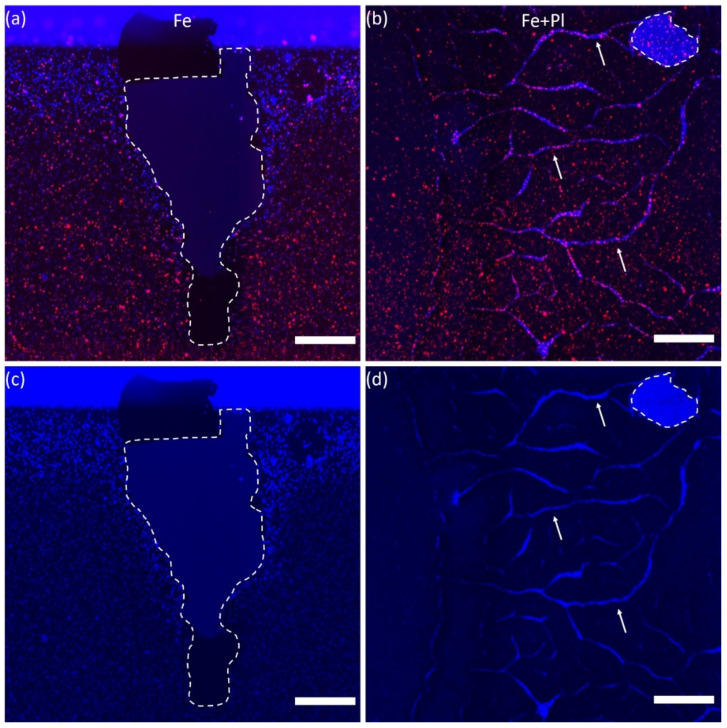
Representative fluorescence images of β-tubulin III labelled neurons (red) and cell nuclei (blue) for the Fe (**a**) and Fe+Pl (**b**) preparations showing a large delaminated area on the edge of an Fe sample where no cells can be seen, and both cracks and delamination on an Fe+Pl sample (**b**,**d**). (**c**,**d**) Images showing only the DAPI staining in the regions depicted in (**a**) and (**b**), respectively. The delaminated areas are indicated by the dashed lines and some examples of cracks by the arrows. Scale bars: 500 µm.

Notably, VACNTs grown without the Al underlayer (Fe and Fe+Pl preparations) delaminated from the substrate, and in some cases already during sample handling (Figure S3a, these samples were not used in the cell cultures). In preparations lacking the Al underlayer, delamination was seen to occur also after the culture and immunohistochemistry procedures and even during cell culture in samples that appeared otherwise intact at the start of the cell culture (Figure 2 or Figure S3b,c). In the example shown in Figure 2a,c, the VACNT film is seen to have delaminated from the substrate at the edge of a Fe preparation. The fact that no cells can be seen in the delaminated area indicates that the detachment of the CNTs occurred following culture and processing of the cells on the sample. In the Fe and Fe+Pl preparations, delamination occurred also during cell culture (Figure 2 and Figure S3b,c). Using higher magnification, areas were found in these samples where cells were at a different focal plane from cells in the rest of the sample. This suggests that delamination occurred during the process of cell seeding or shortly thereafter and that cells in these delaminated areas adhered to the Si surface rather than to the tips of the VACNTs. In the Fe+Pl preparation there were, in addition to delaminated areas, visible cracks on the surface of the samples (Figure 2b,d). On the other hand, addition of the Al underlayer stabilized the film and prevented the VACNTs from cracking or delaminating from the surface. CNT stability of the four preparations was also quantified by measuring the total VACNT delamination and crack area and normalizing by the total available area per sample. The cracks and delaminated areas were identified as areas that had higher reflected intensity in the DAPI channel compared to the CNT covered surfaces. The results, given as the average delaminated area ± the standard deviation across each preparation, showed that the Al/Fe and Al/Fe+Pl preparations were the most stable with close to zero delamination or cracks observed on the surface (there were no Al/Fe samples with delaminated areas and only one Al/Fe+Pl sample had a 0.01% normalized delamination area). They were followed by the Fe preparation with a delamination area of 0.1 ± 0.002% after immunohistochemistry, and finally the Fe+Pl preparation was the least stable with a delamination area of 6.7% ± 0.04%.

High-resolution TEM characterization of our VACNT material showed clear sidewalls and a hollow core, demonstrating that our process synthesized multi-walled CNTs (MWCNTs) (Figure 1b). MWCNTs are metallic, so the VACNTs produced here meet the most basic electrical requirement—they conduct electricity. The nanotube diameters were 10–15 nm. TEM also revealed the presence of nanoparticle impurities encapsulated within the nanotubes; we did not observe any nanoparticles on the surfaces of the nanotubes. Because of the high contrast and size of these nanoparticles, they are composed of atoms that are much heavier than carbon [53], and they are most likely Fe catalyst nanoparticles. Since transition metal catalysts are known to be cytotoxic [54], this encapsulation should prevent the release of these nanoparticles, thereby improving the biocompatibility of our VACNT preparation. TEM also showed the inner CNT walls are highly ordered, and crystalline. Raman spectroscopy confirmed the CNTs possess long range order and sp^2^ crystallinity (SM). However, the outer few walls and the surface of the nanotubes became defective and amorphous. Part of the surface disorder could be amorphous carbon residue left over from TEM sample preparation, but the presence of a defect peak in the Raman data indicates that at least some of the disorder is innate to the as-grown nanotube (Figure S4). Finally, surface hydrophobicity plays an important role in neurite outgrowth [12]. The VACNTs had WCAs of 158.7 ± 5.1° for the Fe preparation and 158.3 ± 2.6° for the Al/Fe preparation, indicating that both preparations were superhydrophobic (Figure S5). The Fe+Pl and Al/Fe+Pl preparations were superhydrophilic; the water instantaneously wet the entire substrate so that the WCA could not be determined.

**Figure 3:**
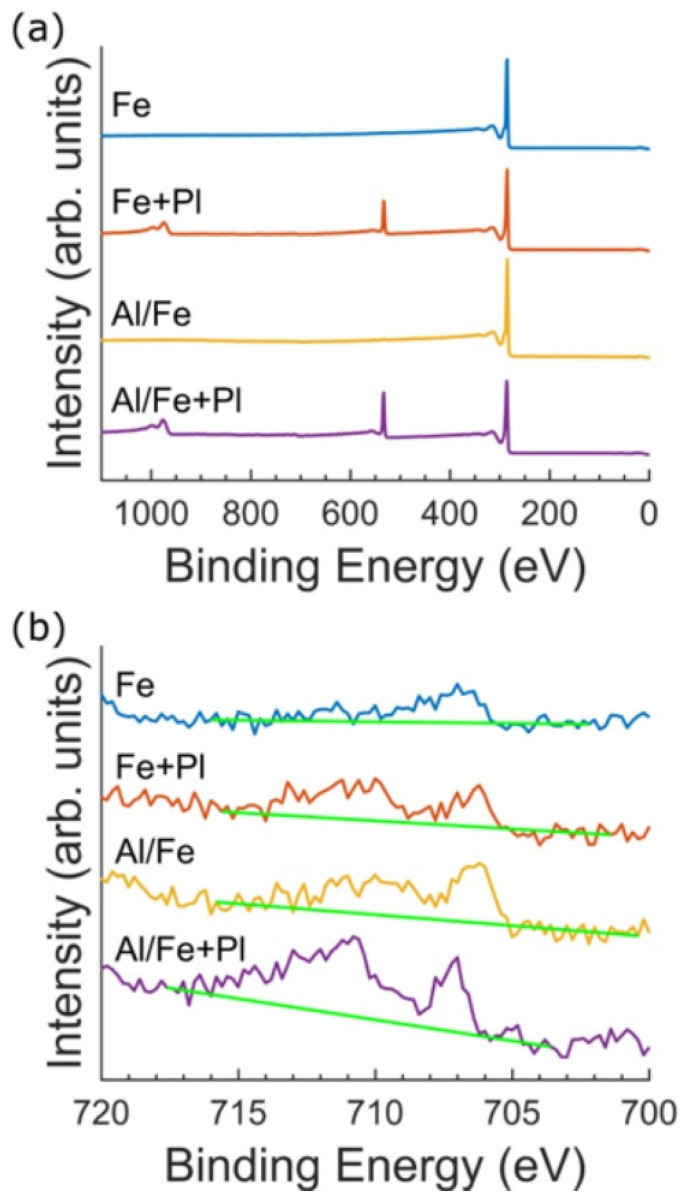
XPS survey scans for all four VACNT preparations. (**a**) XPS survey scans for the Fe (blue), Fe+Pl (red), Al/Fe (yellow), and Al/Fe+Pl (purple) preparations. All preparations clearly feature the C 1s peak at 284.8 eV. Additionally, the oxygen functionalized preparations feature an O 1s peak near 533 eV. (**b**) XPS Fe 2p_3/2_ scans. Background signal is shown for each scan in green. For reference, the binding energy of metallic Fe is 706.7 eV and that of Fe_2_O_3_ is 710.8 eV.

In order to identify potential cytotoxic compounds present in the VACNT forests, we obtained the elemental composition of the forests by using XPS. Because XPS is only sensitive to the outermost 10 nm of the sample, it identifies elements that may be in direct contact with the neurons and glia. XPS survey spectra from all four preparations are dominated by the C 1s peak. Two peaks were used to fit the C 1s peak envelopes: an asymmetric main peak arising from the graphitic sp^2^ C=C bonds and a second peak to fit the characteristic π–π* shakeup feature near 291 eV (SM) (Figure S6). The C spectra are consistent with the bond chemistry of high-purity CNTs (99.6–99.9%). For the Fe and Al/Fe preparations, the O content was either below detection limits or extremely low (Figure 3a). The Fe+Pl and Al/Fe+Pl preparations featured an O 1s peak occurring near 533 eV, which is standard for plasma functionalized VACNTs [49,50]. The high-resolution spectra of the Fe 2p_3/2_ region are shown in Figure 3b. The low binding energy feature in the Fe 2p_3/2_ spectra appears at a binding energy characteristic of metallic Fe (706.7 eV), while the feature near 710 eV in the spectrum from the Al/Fe, Fe + Pl, and Al/Fe+Pl preparations may indicate the presence of an oxide, although the atomic percentage is too low to be definitively resolved. The total atomic percentage of Fe was similar for all preparations: 0.1% for the Fe, Al/Fe, and Fe+Pl preparations, and 0.2% for the Al/Fe+Pl preparation. The 10 nm information depth provided by XPS indicates the Fe is either trapped within the VACNTs or exposed at the VACNT surface. No other elements, including Al, were detected by XPS, suggesting that Al does not migrate from the substrate surface during VACNT synthesis.

**Figure 4:**
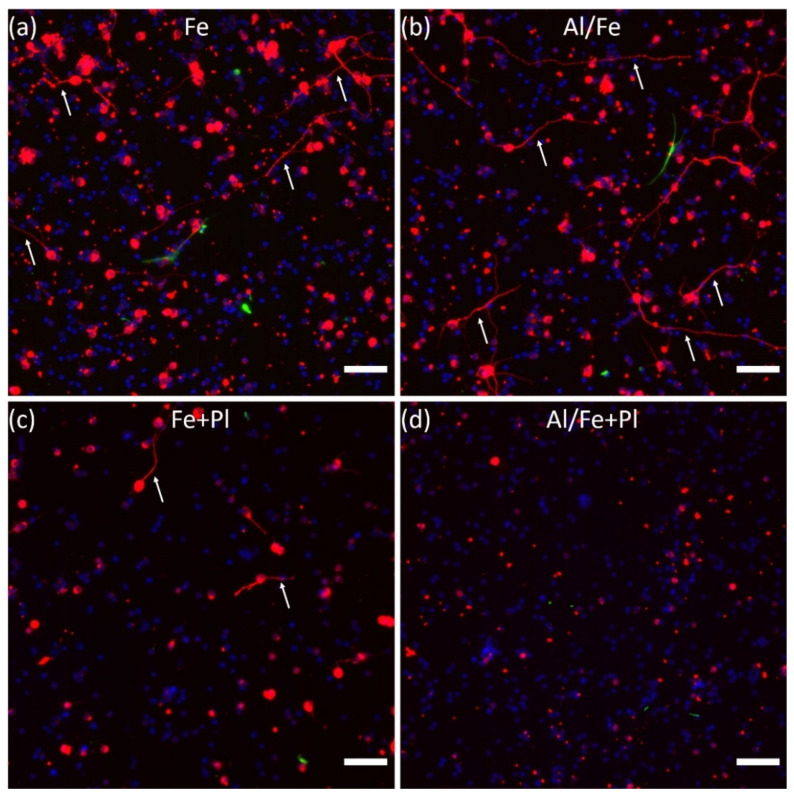
Representative fluorescence images of β-tubulin III labelled (red) neurons and neurites (arrows), GFAP labeled glia (green), and cell nuclei (blue) on all VACNT preparations. (**a**) Fe, (**b**) Al/Fe, (**c**) Fe + Pl, and (**d**) Al/Fe+Pl showing the occurrence of neurite-bearing cells and the extent of neurite outgrowth in the different preparations. Scale bars: 50 µm.

**Figure 5:**
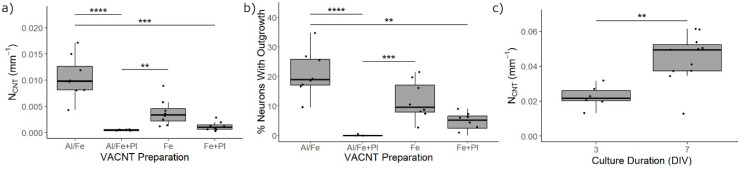
Statistical comparison of the normalized neurite length and percentages of neurons with outgrowths. (**a**) Uniform samples. The median normalized neurite length after 3 days in vitro (DIV) is greatest for neurons grown on the Al/Fe preparation. Statistical analysis revealed that the Al/Fe preparation was significantly greater than the Al/Fe+Pl and Fe+Pl preparations. (**b**) Uniform samples. The percentage of neurons with neurite outgrowths after 3 DIV is greatest for neurons grown on the Al/Fe preparation. Statistical analysis revealed that the Al/Fe preparation was significantly greater than the Al/Fe+Pl and Fe+Pl preparations. (**c**) Patterned samples. The median normalized neurite length was seen to have a statistically significant increase between 3 and 7 DIV, demonstrating the biocompatibility of the Al/Fe group at longer culture times. Each block dot represents the average value for one of the samples. ****, ***, and ** correspond to *p* values <0.0001, <0.005, and <0.01 respectively.

We assessed the biocompatibility of group one VACNT forests with retinal neurons by measuring the extent of neurite outgrowth and the percentage of neurite-bearing neurons. We found that the Fe, Al/Fe, and Fe+Pl preparations supported neuronal process outgrowth for at least 3 DIV (Figure 4a–c), while the Al/Fe+Pl preparation showed little to no neurite growth (Figure 4d). For the Fe, Al/Fe, and Fe+Pl preparations, neurites of several hundred microns were observed. The morphology of the glial cells was similar across the Fe, Al/Fe, and Fe+Pl preparations (Figure 4a,b, or Figure S3c), but no glial cells were observed for the Al/Fe+Pl preparation. In order to quantify neurite outgrowth, we compared the normalized neurite lengths among the preparations. Neurites were successfully extracted using our automated image analysis (Figure S7). We found an error of <5% in the neurite length extracted by the automated algorithm compared to neurite lengths extracted using the semi-automated ImageJ plugin Simple Neurite Tracer on 5 different images. Kruskal–Wallis analysis with post-hoc Dunn’s testing revealed the Al/Fe preparation gave the largest median normalized neurite length (Figure 5a), with it being about three times larger than the Fe preparation. Though no statistical difference in neurite outgrowth was found between the Fe and Al/Fe preparations, the Al/Fe preparation performed significantly better than the two plasma treated preparations in neurite outgrowth (Figure 5a). Kruskal–Wallis analysis with post-hoc Dunn’s testing results for the percentage of neurons with outgrowth revealed that the Al/Fe preparation performed significantly better than the two plasma treated preparations. Although no significance was detected between the Fe and Al/Fe preparations, the median of the percentage of neurons with outgrowth was about two times larger than the Fe preparation (Figure 5b).

Having observed the superior mechanical stability of the Al/Fe preparation as well as its ability to promote neurite outgrowth during the biocompatibility test, we moved to the second group (patterned 100 µm wide VACNTs) to determine how neurite outgrowth would change during longer cultures and demonstrate that these VACNTs can be patterned to achieve the desired geometric properties of a retinal implant electrode. As it has been shown that the height and structure of VACNTs can be affected by the size and geometry of the catalyst patterning [55], the width of the second group was fixed at 100 µm to ensure that neurons were interfacing with VACNTs with aspect ratios that provided robustness, similar heights, and sufficiently large surfaces (an order of magnitude larger than a cell body) with similar textures to the first group (uniform VACNTs). Since the texture of surfaces interacting with neurons is known to affect neurite outgrowth [39], we chose to maintain a consistent texture between culture groups. Kruskal–Wallis analysis of the median normalized neurite length on this patterned group showed a statistically significant increase between 3 and 7 DIV (Figure 5c).

In order to achieve a high visual acuity, future subretinal implants would benefit from electrode geometries with a high-aspect-ratio, an electrode component width as small as a few micrometers, and a separation between these components that is also as small as a few micrometers. We demonstrate that VACNTs grown from the Al/Fe preparation can provide these benefits. The VACNTs in the third group have row widths and row spacings ranging from 2.5 to 20 µm and heights ranging from 15 to 20 µm (Figure S1). Rows with a 2.5 µm width, 10 µm spacing, and 20 µm height are pictured in Figure 1d. Thus, we have demonstrated that our VACNT growth and patterning process is potentially viable for high visual acuity subretinal implants.

## 4. Discussion

We investigated whether the addition of an adhesive Al layer resulted in improved mechanical integrity of VACNTs and the extent to which the addition of this layer affected biocompatibility. We cultured dissociated retinal cells on four different VACNT preparations with and without the Al layer, and with and without oxygen plasma functionalization. No functionalization with ECM components was used prior to or during the culture, as this would have altered the surface characteristics of our VACNTs. Our goal was not to actively promote cell adhesion and/or survival, but rather study the interactions of retinal neurons with the as-produced VACNT surfaces and the stabilities of these different VACNT preparations over time in culture. The median normalized neurite length and median percentage of neurons with outgrowth on VACNTs prepared from an Al underlayer with the Fe catalyst (Al/Fe preparation) improved about three and two times, respectively, compared to VACNTs prepared without the Al underlayer (Fe preparation). This improvement in both parameters for the Al/Fe preparation compared to the Fe preparation occurred despite the presence of a small atomic percentage of Fe (0.1%) and possible Fe_2_O_3_ at the CNT top surface, as measured by XPS in both preparations. Previous research has shown Fe_2_O_3_ nanoparticles reduce neural cell viability three days post-exposure by ~25% for Fe_2_O_3_ nanoparticle concentrations of 1.5 mM and by ~90% for concentrations of 15 mM [56]. Because of the observed neurite outgrowth, it is therefore likely that the Fe or Fe_2_O_3_ catalyst nanoparticles never encounter the cells for the Fe and Al/Fe preparations. This was supported by the significant increase in neurite outgrowth seen between the 3 and 7 DIV cultures on our patterned Al/Fe samples. Furthermore, TEM imaging revealed that the CNTs encapsulate non-carbon, heavy-atom nanoparticles within their many walls. These nanoparticles were not observed on the outer surface of the nanotubes. Combining the TEM and XPS results, we conclude the encapsulated nanoparticles are the Fe-based catalysts. This encapsulation provides a means to separate the cells from potentially cytotoxic nanoparticles, and therefore contributes greatly to the biocompatibility of VACNT preparations without plasma functionalization up to at least 7 DIV.

Our results established that the Al/Fe and Fe preparations have similar nanotopographies, hydrophobicities, and atomic compositions. In fact, the only major difference we detected was that the Fe preparation delaminated easily from the substrate (Figure 2 or Figure S3), while the Al/Fe preparation did not. The reductions in neurite outgrowth and percentage of neurons with outgrowth in the Fe preparation may be due to exposure to CNTs dispersed in the cell culture medium. In a previous study investigating the effect of dispersed MWCNTs on axonal regeneration of mouse dorsal root ganglia [46], incubation with MWCNTs (10–20 µm in length prepared from Fe catalyst) at concentrations increasing from 1 µg/mL to 10 µg/mL caused a reduction in regenerated axon length by 40% or 70%, respectively, compared to a control. However, these concentrations did not cause cell death. We hypothesize that CNTs with the Fe preparation detach either before and/or during the culture, leading to a similar reduction in neurite length without cell death. In particular, our calculation shows that for VACNTs of height 30 µm on a 1 cm^2^ chip in 500 µL of cell suspension, as prepared here, along with a graphitic density of 2.1 g/cm^3^, CNT dislodgement at the level of 0.1% by weight (i.e., 10 µg/mL) could cause the ~65% reduction in median normalized neurite length between the Fe and Al/Fe preparations. Since there was no significant difference in neuron density between the two preparations (Figure 4a,b for visual comparison, statistical data not shown), the increase in normalized neurite length in the Al/Fe preparation occurs because of a more favorable growth environment.

An unexpected result from our research is that plasma functionalization of the VACNTs, to make them more hydrophilic, reduced both neurite outgrowth and the percentage of neurons exhibiting neurite outgrowth. In contrast, previous research on plasma-functionalized, short (~2 µm), sparse VACNTs prepared from a Ni catalyst showed that hippocampal neurite outgrowth was greater than on the unfunctionalized, adsorptive, and covalently modified surfaces [31]. On horizontally-oriented, solution-deposited CNTs functionalized with carboxylic and hydroxide groups, dorsal root ganglia extended long neurites which strongly interconnected with the underlying CNT surface [57]. Plasma functionalization of vertical CNTs prepared from a Fe catalyst has also been shown to increase adhesion and the cell spreading area of fibroblasts [58]. A hypothesis for the lack of neurite outgrowth seen in our Fe+Pl and Al/Fe+Pl preparations in the present study is that the Fe_2_O_3_ nanoparticles that are trapped in the CNT tips are freed during plasma etching. Previous research has found that oxygen plasma functionalization of CNTs grown from a Fe catalyst with an Al underlayer does indeed open the tips of some nanotubes, but does not affect their structure [49]. Coupling this with the known dose dependent toxicity of Fe_2_O_3_ nanoparticles after 3 days post-exposure [56], the lack of neurite outgrowth may indicate a larger concentration of Fe_2_O_3_ nanoparticles is present in the Al/Fe+Pl preparation versus the Fe+Pl preparation.

Aside from biocompatibility concerns, electrodes interfacing with neuronal networks must be physically stable and mechanically strong to provide support for the cells [59]. Although the fabrication of the VACNTs grown without the Al underlayer (Fe preparation) entails fewer fabrication steps, the VACNTs on both the Fe and Fe+Pl preparations easily delaminated from the substrate during handling and culture of the samples, thereby indicating that these preparations do not possess the mechanical stability necessary for in vivo applications (Figure S3). The addition of the Al underlayer stabilized the VACNT film, and in the case of the Al/Fe preparation, yielded favorable neurite outgrowth up to 3 DIV when compared with the Fe preparation. Furthermore, the patterned Al/Fe samples supported neurite outgrowth up to 7 DIV. Therefore, the addition of the Al underlayer constitutes a viable approach for the development of electrodes that may ultimately be used in in vivo studies.

We were motivated to test the role that an Al underlayer plays in the biocompatibility of VACNTs with retinal cells because VACNTs satisfy the myriad material and geometric requirements for electrodes in high visual acuity retinal implants. In terms of geometric requirements, VACNTs are an excellent candidate because in principle they can be patterned into high-aspect-ratio structures while achieving a minimum feature size and spacing approaching 1 µm. Here, in our preliminary studies, we have shown that our VACNT synthesis process can be combined with catalyst patterning to produce VACNT rows with widths and spacing down to 2.5 µm and with heights of 20 µm. Therefore, our VACNT process can generate the electrode structures needed for high visual acuity retinal implants. High-aspect-ratio VACNT electrodes could (1) potentially reduce glial scar formation, as has been observed in other high-aspect-ratio devices [23,24] and (2) reduce the electrode thresholds for inducing membrane potential changes in bipolar neurons [9,26,27]. Due to the relatively low temperature (650 °C) used here to prepare the VACNTs, we expect the fabrication procedure outlined would be compatible with semiconductor processing used to fabricate current generation subretinal implants [4,60].

A comprehensive study of the VACNT-substrate adhesion forces and their dependence on catalyst conditions, and the plasma functionalization parameters (such as power and duration) and their effects on Fe and CNT concentrations in the culture medium is needed in order to achieve the most suitable VACNT environment for enhancing neuronal survival and neurite outgrowth. Future work will include studying the effects of various functionalization methods, the electrochemical properties of the as-produced VACNTs, and the longer in vitro dissociated retinal cell and explant cultures, and in vivo studies using animal models of photoreceptor degeneration. In addition, VACNTs will either be transferred to or directly grown on flexible substrates to reduce in vivo adverse tissue responses.

## 5. Conclusions

In conclusion, we produced large-area, uniform VACNTs and high-aspect-ratio, micrometer-size VACNT structures by using a CVD growth process on Fe and Al/Fe catalysts. Without any functionalization, the VACNT forests supported retinal neurite growth up to at least 7 DIV. Interestingly, plasma functionalization of VACNTs grown from Al/Fe catalysts led to a significant loss in neurite outgrowth and a reduced percentage of neurons with outgrowth, indicating that careful optimization of plasma functionalization protocols must be undertaken for creating biocompatible VACNT surfaces. The Al/Fe preparation led to VACNTs with increased mechanical stability and more extensive neurite outgrowth. These results demonstrate that our VACNTs are biocompatible with primary retinal neurons. Furthermore, by controlling the patterning and height of the VACNTs, we have opened up the possibility of exploring geometric enhancements to neural stimulation with a CNT electrode by reducing, for instance, glial scar formation and voltage thresholds, and decreasing overall pixel size. Because we demonstrated robust neurite outgrowth without functionalization, it may also be possible to provide further improvements through chemical modifications of the CNT surface. Taken together, VACNT electrodes synthesized from an Al/Fe catalyst multilayer represent an option well worth pursuing to further improve the vision restoration provided by modern retinal implants.

## Supplementary Materials

The following are available online at https://www.mdpi.com/2072-666X/11/6/546/s1. CNT additional characterization information, including transmission electron spectroscopy, Raman spectroscopy, and X-ray photoelectron spectroscopy. Figure S1: SEM images group three showing high-aspect-ratio VACNTs. Figure S2: Top view and 30° tilted SEM images of the four preparations in group one (Fe, Al/Fe, Fe+Pl and Al/Fe +Pl). Figure S3: SEM and fluorescence images showing VACNT delamination and cracking pre and post-culture. Figure S4: Raman spectroscopy for characterizing the as-grown CNTs prepared from Fe and Fe/Al catalysts. Figure S5: Hydrophobicity test using wetting contact angle goniometry. Figure S6: XPS spectra at the C 1s peaks (left column) and O 1s peaks (right column). Figure S7: Automatically extracted neurites (red) on the Al/Fe image shown in Figure 4b in the results section.
