# Supplementary material for: The Roles of an Aluminum Underlayer in the Biocompatibility and Mechanical Integrity of Vertically Aligned Carbon Nanotubes for Interfacing with Retinal Neurons
Source: Micromachines (Basel). 2020 May 28;11(6):546. doi: 10.3390/mi11060546 (PMC7345717; doi:10.3390/mi11060546)
Supplement: Supplementary file 1 [file micromachines-11-00546-s001.pdf]

Supplementary Materials: The Roles of an Aluminum Underlayer in the Biocompatibility and Mechanical Integrity of Vertically Aligned Carbon Nanotubes for Interfacing with Retinal Neurons

William J. Watterson, Saba Moslehi, Conor Rowland, Kara M. Zappitelli, Julian H. Smith, David Miller, Julie E. Chouinard, Stephen L. Golledge, Richard P. Taylor, Maria-Thereza Perez and Benjamín J. Alemán

CNT Additional Characterization Information

Transmission Electron Spectroscopy

VACNTs were imaged at 80kV with a FEI Tecnai Transmission Electron Microscope. VACNTs grown using the methods described in the main text were scraped off of a ~1cm^2 silicon/silicon dioxide wafer into ~1 ml of isopropanol. The isopropanol/CNT solution was then sonicated for ~2 hours. After sonication, 7μl of solution was dropped onto a suspended TEM grid (Ted Pella Lacey Carbon, 400 mesh Au) and let dry overnight.

Raman Spectroscopy

Raman spectroscopy was performed to characterize the as-grown VACNT samples using a WITEC alpha300 Raman microscope with a 532 nm excitation laser, a 20× objective, and a 600 g/mm diffraction grating. Raman spectra were normalized to the height of the silicon peak at ~520 cm^{-1} for comparison.

X-Ray Photoelectron Spectroscopy

The binding energy scales for the high-resolution spectra were calibrated by setting the main feature of the carbon (C) 1s peak envelope to 284.8 eV. The balance of the composition was attributable to C. Survey spectra were acquired at a pass energy of 150 eV. High resolution spectra of Fe 2p, C 1s, O 1s, and Al 2p regions acquired at a pass energy of 20 eV were used to determine the composition. Fe 2p spectra were obtained by averaging over 75 scans.

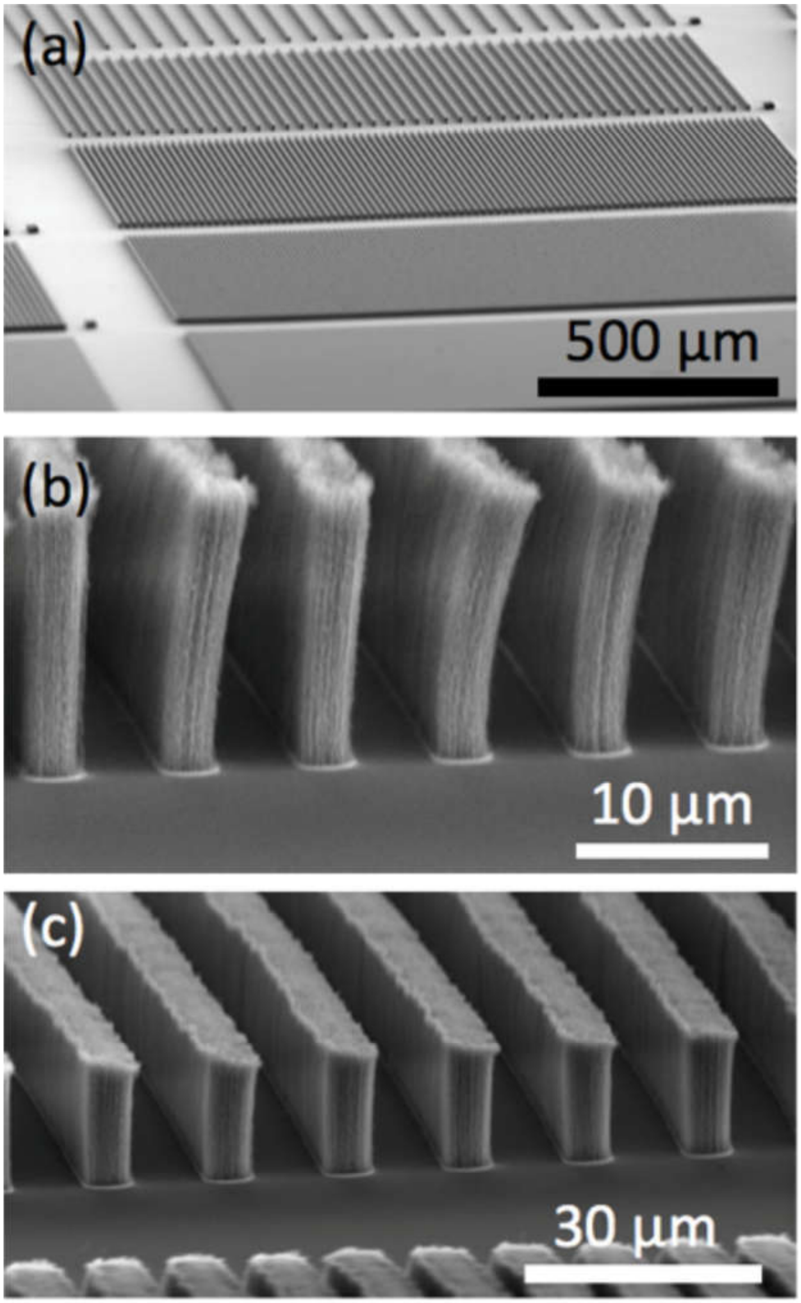

Figure S1. SEM images of group three showing high aspect ratio VACNTs. (**a**) VACNT rows of varying row width and row spacing. (**b**) VACNTs with a row width of 2.5 µm, a row spacing of 5 µm, and a height of 15 µm. (**c**) VACNTs with a row width of 5 µm, a row spacing of 10 µm, and a height of 15 µm.

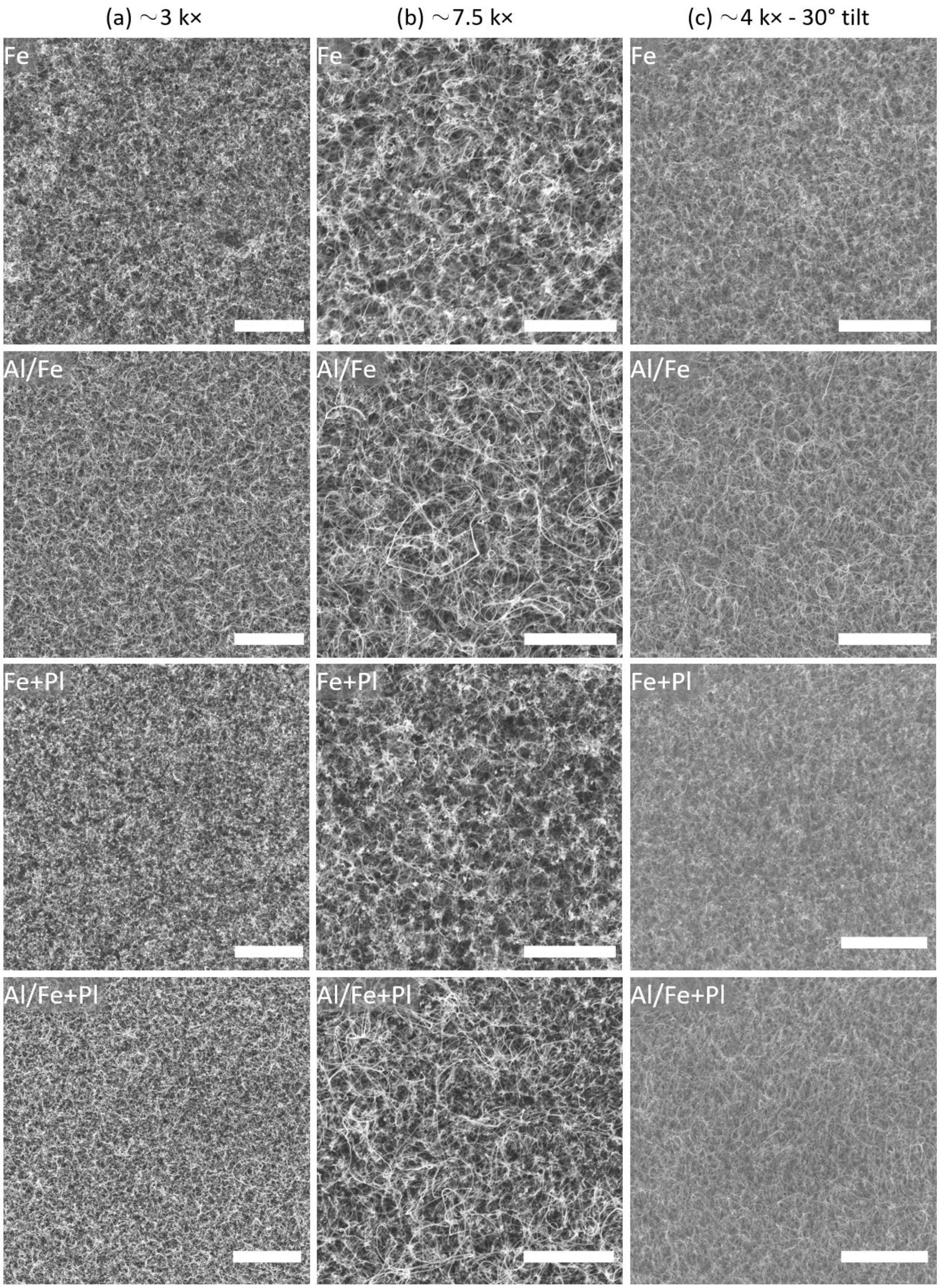

Figure S2. Top view and 30° tilted SEM images of the four preparations in group one (Fe, Al/Fe, Fe+Pl and Al/Fe +Pl). Column (**a**) ~3 k× magnification, top view; column (**b**) ~7.5 k× magnification, top view; column (**c**) ~4 k× magnification, 30° tilted view. Scale bars: 6, 3 and 6 μm for SEM images in columns (**a**), (**b**) and (**c**), respectively.

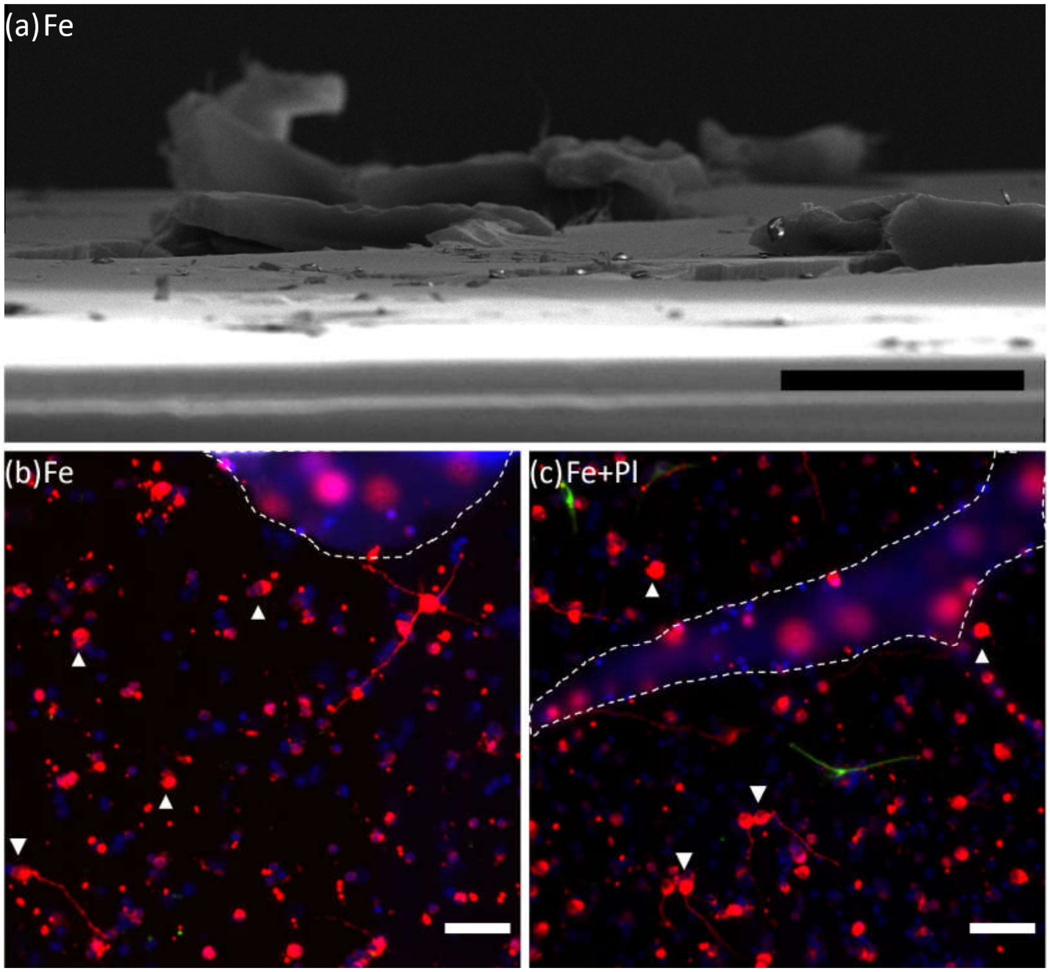

Figure S3. SEM and fluorescence images showing the effect of VACNT delamination and of cracking pre and post- culture. (**a**) A 90° tilted SEM image of an Fe sample taken before the cell culture that clearly shows delamination of the CNT layer from the substrate (sample was not used in a cell culture experiment). Scale bar: 300 μm. (**b**) and (**c**) Representative fluorescence images of neurons (red), glia (green) and cell nuclei (blue) for the Fe (**a**) and Fe+Pl (**b**) preparations showing a delaminated area on the Fe and a crack with delamination on the Fe+Pl preparation respectively (indicated by the dashed lines). Within the delaminated areas, cells and their nuclei appear blurred as they are not at the same focal plane as cells visible on top of the VACNTs (arrowheads). Scale bar: 50 μm.

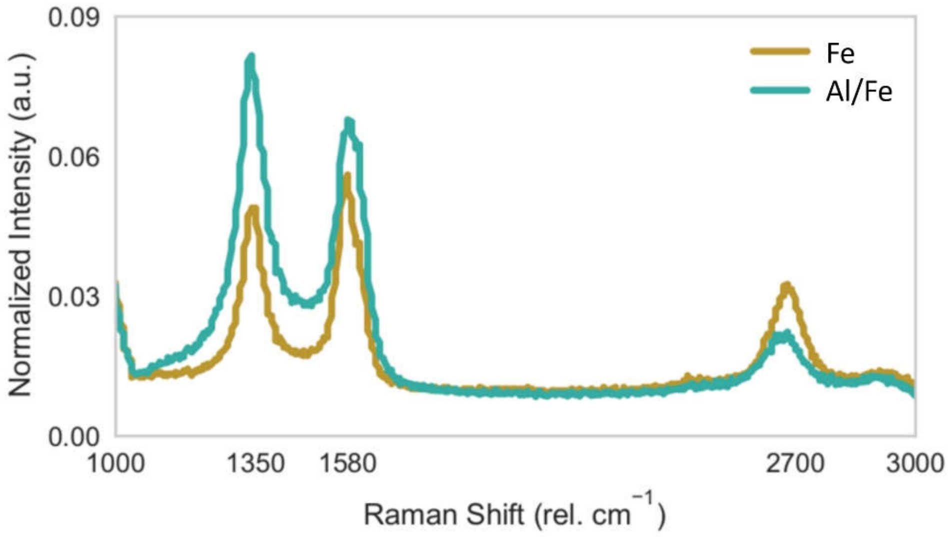

Figure S4. Raman spectroscopy for characterizing the as-grown VACNTs from the Fe and Al/Fe preparations. Three characteristic peaks are observed related to the presence of carbon nanotubes, the D-peak at ~1350 cm^{-1}, which is due to sp^3 bonding in amorphous carbon impurities or broken sp^2 bonds in the lattice, the G-peak at ~1580 cm^{-1}, which arises from high crystallinity in the sample, and the G′ peak at ~2700 cm^{-1}, which is characteristic of long-range order and crystallinity. The relative intensities of these peaks can be used to give a qualitative assessment of the purity of a CNT sample [1], however, the lack of a 100% pure CNT sample grown using our methods precludes a quantitative determination of the purity. We find that both the Fe and Al/Fe preparations have a G′ peak, which shows that our CVD grown samples are indeed MWCNTs. The high ratio of D/G intensities in both the Fe and Al/Fe preparations indicates that there are some degree of lattice defects and amorphous carbon contamination.

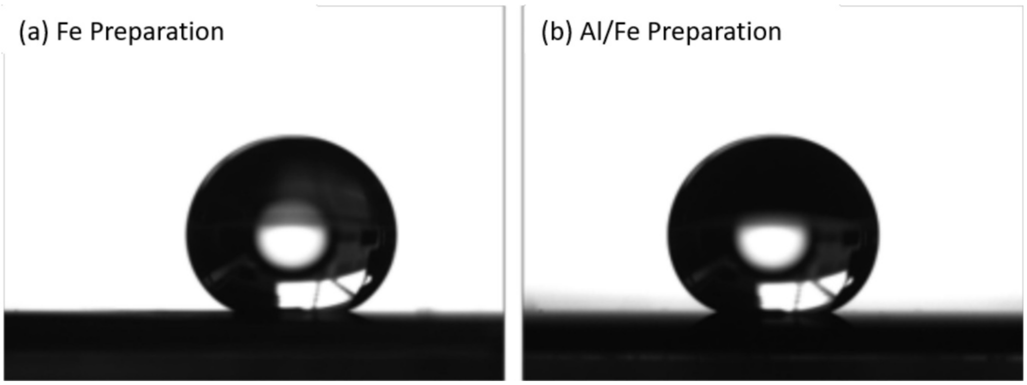

Figure S5. Hydrophobicity test using wetting contact angle goniometry. Wetting contact angle measurements for 10 μL water drops on (**a**) the Fe and **(b**) Al/Fe preparations are 158.7 ± 5.1° and 158.3 ± 2.6°, respectively.

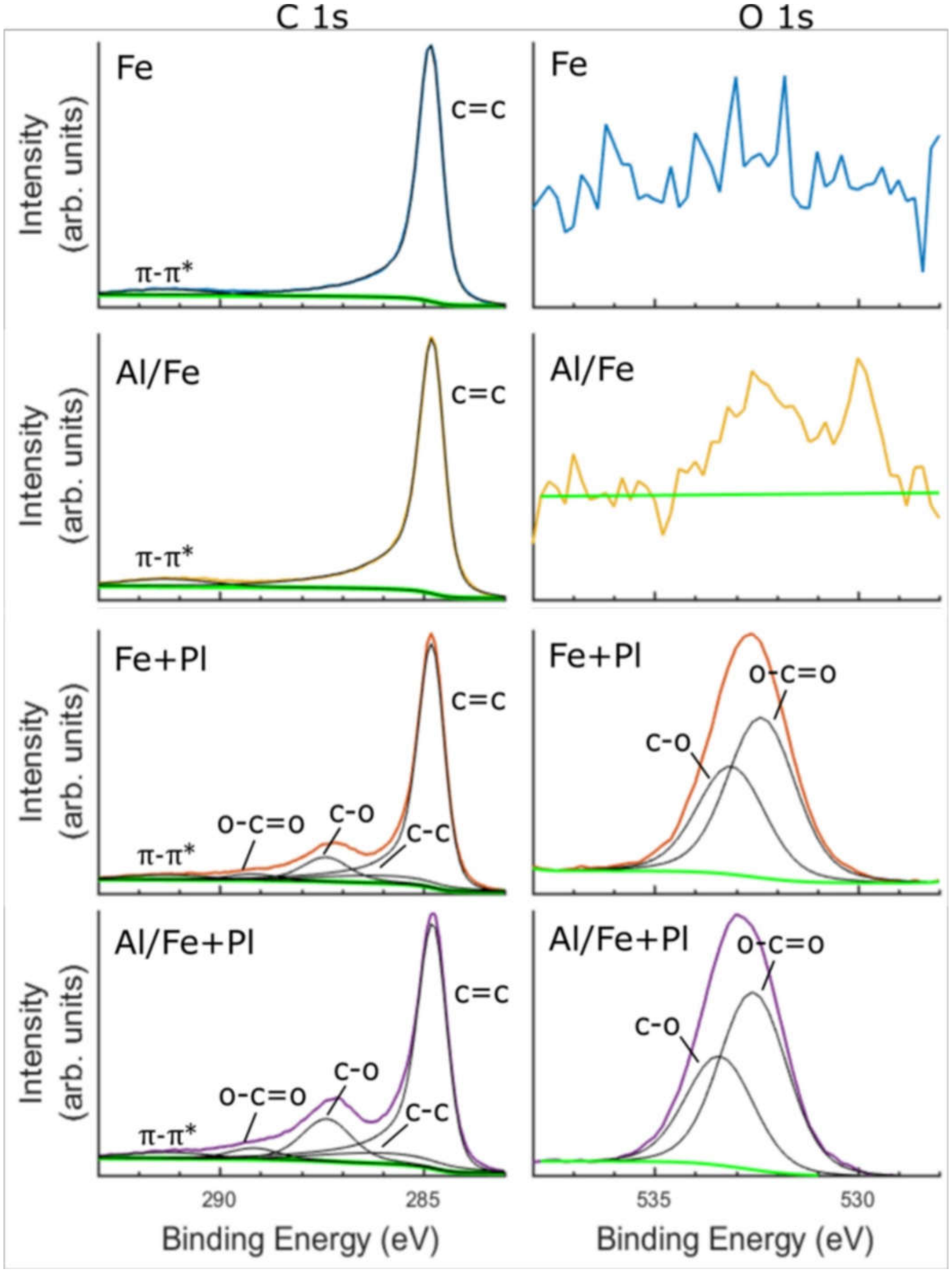

Figure S6. XPS spectra at the C 1s peaks (left column) and O 1s peaks (right column). Each spectrum is associated with the CNT surface indicated by the preparation name in the top left. Background signal is shown in green. C 1s peak deconvolution showed graphitic sp^2 C=C and π-π* bonds for both surfaces. No oxygen was detected for the Fe preparation and only a minimal amount (0.3%) for the Al/Fe preparation. The feature in the Al/Fe O 1s spectrum near 530 eV is consistent with oxygen in an iron oxide while the 532.5 eV feature is consistent with oxygen bound to carbon.

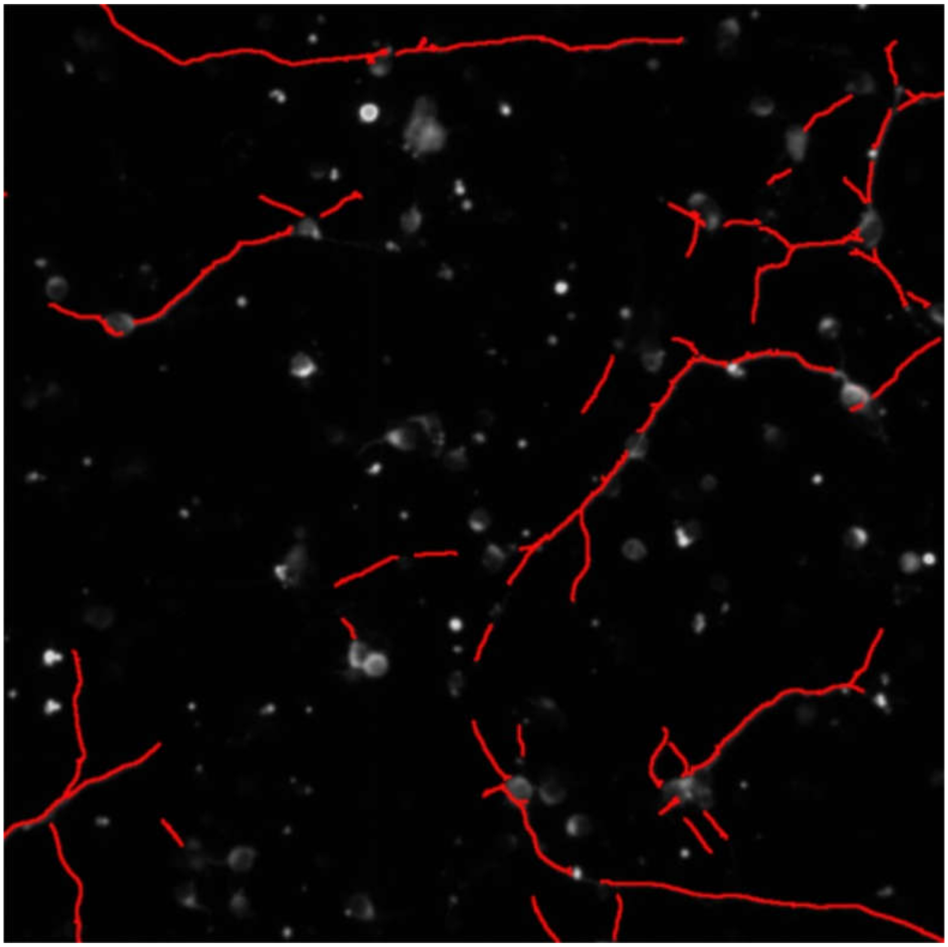

Figure S7. Automatically extracted neurites (red) on the Al/Fe image shown in Figure 3b in the results section.

References

1. DiLeo, R. A.; Landi, B. J.; Raffaelle, R. P. Purity Assessment of Multiwalled Carbon Nanotubes by Raman Spectroscopy. *J. Appl. Phys.* **2007**, *101*, 064307. https://doi.org/10.1063/1.2712152.
